# Effects of dietary *Bacillus velezensis* fermented soybean hull supplementation on antioxidant capacity, suppressing pro-inflammatory, and modulating microbiota composition in broilers

**DOI:** 10.1016/j.psj.2025.104827

**Published:** 2025-01-23

**Authors:** Yung Hao Chen, Yi Chen Li, Shen Chang Chang, Min Jung Lin, Li Jen Lin, Tzu Tai Lee

**Affiliations:** a Department of Animal Science, National Chung Hsing University, Taichung 40227, Taiwan; b Professional Master Program of Agricultural Business Management, National Chung Hsing University, Taichung 40227, Taiwan; c Southern Region Branch, Taiwan Livestock Research Institute, Ministry of Agriculture, Executive Yuan, Pingtung 91201, Taiwan; d Bachelor of Program in Scientific Agriculture, National Pingtung University of Science and Technology, Pingtung 91201, Taiwan; e School of Chinese Medicine, College of Chinese Medicine, China Medical University, Taichung 40402, Taiwan; f The iEGG and Animal Biotechnology Center, National Chung Hsing University, Taichung, 40227, Taiwan; g Smart Sustainable New Agriculture Research Center (SMARTer), Taichung 40227, Taiwan

**Keywords:** Fermented soybean hull, *Bacillus velezensis*, Intestinal microbiota, Broilers

## Abstract

This study aimed to ferment soybean hulls (**SBH**) with *Bacillus velezensis* and evaluate their effects on broiler diets, specifically focusing on intestinal antioxidant capacity, immune modulation, and microbiota composition. The animal trial involved 400 one-day-old Arbor Acres broilers, randomly assigned to a control group (basic diet, Control) and groups receiving 5 % and 10 % unfermented soybean hulls (5 % **USBH**, 10 % USBH) and 5 % and 10 % fermented soybean hulls (5 % **FSBH_B_**, 10 % FSBH_B_) as replacements for the basic diet. Each group contained 80 birds, divided into four pens with 20 birds per pen, and the trial lasted for 35 days. In the jejunum, the 5 % FSBH_B_ group tended to suppress pro-inflammatory gene expression, while the 10 % FSBH_B_ group tended to enhance antioxidant gene expression. In terms of jejunum protein levels, the 10 % FSBH_B_ group exhibited significantly lower (*P* < 0.05) TNF-α protein levels compared to the control and other treatment groups. Furthermore, intestinal microbiota analysis showed that ileum and cecum microbial counts in the 10 % USBH and 10 % FSBH_B_ groups were higher than those in the control group. Species richness indices also revealed that both the 10 % USBH and 10 % FSBH_B_ groups were significantly higher (*P* < 0.05) than the control group. In conclusion, soybean hulls fermented with *Bacillus velezensis* improved intestinal antioxidant capacity, suppressed pro-inflammatory gene expression, and modulated microbiota composition in broilers, with the 10 % FSBH_B_ group demonstrating the most pronounced effects.

## Introduction

In recent years, the total production of soybeans has gradually increased to meet the growing demand for soy products. According to the United States Department of Agriculture (**USDA**) World Agricultural Production data, the total soybean production in 2023/2024 reached 398.9 million tons (USDA, 2023). Brazil, the United States, Argentina, China, and India are the major producers, with Brazil and the United States each accounting for about one-third of global production. During soybean processing, by-products such as soybean meal, soybean dregs, and soybean hulls are produced, with soybean hulls comprising 5-8 % of the total soybean weight (Bittencourt et al., 2021), equating to 19-30 million tons annually. Soybean hulls, as high-fiber agricultural by-products, are commonly used as fiber supplements in ruminant feed(Sessa, 2004) and for other applications such as wastewater treatment (Tummino et al., 2020) and ethanol production (Rojas et al., 2014; Mielenz et al., 2009).

The annual soybean import in Taiwan ranges from 2 to 2.7 million tons, primarily from the United States and Brazil, with 12 % used for feed. Based on the import volume, approximately 130,000-210,000 tons of soybean hulls are produced annually. Most soybean hulls are ground and pelletized for use as fiber supplements in ruminant feed. However, the utilization of soybean hulls remains limited. Sun et al. (2021) highlighted that microbial fermentation of soybean hulls could reduce their fiber content, enhancing their value as feed for monogastric animals. Additionally, studies have shown that soybean hulls contain functional components beneficial to animals, including bioactive peptides, phenolic compounds, and soy isoflavones, which serve as natural antioxidants. The metabolites secreted by *Bacillus velezensis*, similar to those of other *Bacillus* (Khalid et al., 2021), were selected and isolated from mixed feeds and patented in Taiwan patent (Taiwan Patent No. I 725354). In animals, these metabolites exhibit properties such as secreting exogenous enzymes, heat resistance, acid and alkali tolerance, and immune modulation. This study hypothesized that soybean hulls fermented with *Bacillus velezensis* could function as a valuable broiler feed ingredient, enhancing intestinal antioxidant capacity, immune regulation, and microbial composition through increased bioactive components in fermented soybean hulls.

## Materials and methods

### Fermented soybean hull preparation

The laboratory screened *Bacillus velezensis* (Taiwan Patent No. I 725354) and cultured in Luria-Bertani broth at 37°C under aerobic conditions for 24 h. A suitable quantity of soybean hulls was placed in a sterilization bag and moistened with RO water to achieve 60 % moisture content. The mixture was then sterilized at 121°C for 20 min. After cooling, *Bacillus velezensis* was inoculated to a concentration of 10^7^ CFU/g and mixed thoroughly. The inoculated mixture was incubated at 37°C for 96 h. After fermentation, the mixture was dried at 50°C for 24 h, ground into a powder, and stored at 4°C.

### Phenolic acid content

Using a modification of Kareparamban et al. (2013), 1 g of sample was extracted with 10 mL of methanol at room temperature for one hour. After extraction, the mixture was centrifuged at 13000 rpm (26400 xg) for 10 min to separate the supernatant. The supernatant was then dried under nitrogen and reconstituted with 1 mL of methanol. The reconstituted sample was subsequently filtered through a 0.22 µm PVDF membrane to remove particulates. High-performance liquid chromatography (HPLC) was performed using a Hitachi CM5000 system equipped with an ACE Excel 5 C18 column (5 μm, 250 × 4.6 mm). The mobile phase consisted of acetonitrile and 10 % acetic acid (20:80) at a flow rate of 1 mL/min, with the column maintained at 30°C. The injection volume was set at 10 μL, and UV detection was performed at 319 nm. Ferulic acid, p-coumaric acid, chlorogenic acid, and caffeic acid were quantified using standard concentrations of 100, 80, 60, 40, 20, and 10 µg/mL.

### Isoflavone content

Following a modification by Achouri et al. (2005), 1 g of sample was extracted with 10 mL of 80 % methanol at room temperature for four hours. The mixture was then centrifuged at 3000 rpm (1400 xg) for 15 min to isolate the supernatant, which was then dried under nitrogen gas. The dried supernatant was reconstituted with 1 mL of 80 % methanol and filtered through a 0.22 µm PVDF membrane to eliminate any particulates. Isoflavone content analysis was conducted using HPLC with a Hitachi CM5000 system equipped with an ACE Excel 5 C18 column (5 μm, 250 × 4.6 mm). The mobile phase consisted of 1 % acetic acid (solvent A) and 100 % acetonitrile (solvent B), delivered at a flow rate of 0.6 mL/min, with the column maintained at 25°C. The injection volume was set at 10 μL, and UV detection was performed at 260 nm. Daidzin, glycitin, genistin, daidzein, glycitein, and genistein were quantified using standard concentrations ranging from 200 to 10 µg/mL.

### Animals and experimental design

The experiment used 400 one-day-old Arbor Acres broilers with a body weight of 38.2 ± 0.6 g. The study protocols were approved by the Animal Care and Use Committee of National Chung Hsing University (IACUC: 112-002). The broilers were randomly divided into groups fed a corn-soybean meal-based diet (Control) and groups where the basal diet was replaced with 5 % and 10 % unfermented soybean hulls (5 % USBH, 10 % USBH) and fermented soybean hulls (5 % FSBH_B_, 10 % FSBH_B_), respectively, resulting in a total of five groups with 80 chickens each (20 chickens per pen, four replicates). The trial lasted 35 days, from April to May 2023, at the Experimental Husbandry Farm of National Chung Hsing University, with an average environmental temperature of 26±4°C and an average humidity of 60±10 %. Throughout the feeding process, the broilers had free access to food and water. The diet composition adhered to the standards recommended by the NRC (1994). The experimental period was divided into two phases: a starter phase (1–21 d, CP 23.0 % and ME 3200 kcal/kg) and a grower phase (22–35 d, CP 20.0 % and ME 3200 kcal/kg), as shown in Table 1, Table 2. The treatment groups were derived from the control group, with soybean hulls replacing 5 % and 10 % of the corn and soybean meal in the diet. These incremental substitutions aimed to evaluate the feasibility of incorporating soybean hulls into the feed and determine the optimal substitution level.

**Table 1:** Ingredients and calculated analysis (% as fed) of the diet for starters broilers (1–21 days).

Ingredients^1^	Control	5 % USBH	10 % USBH	5 % FSBH_B_	10 % FSBH_B_
**Composition**	———————————–%———————————				
Corn, yellow	41.65	31.16	19.2	31	18.95
Soybean meal (CP-44 %)	39	33.55	15.77	32.3	15
Full fat soybean meal	9.2	18	42.2	19.62	43.18
Soybean oil	3.2	5.4	6	5.2	6
Soybean hull	0	5	10	0	0
Fermented soybean hull	0	0	0	5	10
Fish meal (CP-65 %)	3	3	3	3	3
Monocalcium phosphate	1.43	1.43	1.44	1.44	1.45
Calcium carbonate	1.55	1.49	1.42	1.47	1.45
NaCl	0.34	0.34	0.34	0.34	0.34
_DL_-Methionine	0.35	0.35	0.35	0.35	0.35
Choline-Cl	0.08	0.08	0.08	0.08	0.08
Vitamin premix^2^	0.1	0.1	0.1	0.1	0.1
Mineral premix^3^	0.1	0.1	0.1	0.1	0.1
Total	100	100	100	100	100
**Calculated nutrient levels**					
Crude protein, %	23.02	23.02	23.03	23.04	23.03
ME, kcal/kg	3210	3209	3203	3207	3207
Crude fiber, %	2.77	4.27	5.07	4.20	5.02
Calcium, %	1.03	1.01	1.00	1.01	1.01
Total phosphorus, %	0.67	0.66	0.66	0.66	0.66
Lysine, %	1.28	1.32	1.38	1.32	1.38

1 USBH, unfermented soybean hull; FSBH_B_, fermented soybean hull by *Bacillus velezensis*.

2 Vitamins (premix content per kg diet): Vit. A, 15,000 IU; Vit. D3, 3000 IU; Vit. E, 30 mg; Vit. K3, 4 mg; thiamine, 3 mg; riboflavin, 8 mg; pyridoxine, 5 mg; Vit. B12, 25 μg; Ca-pantothenate, 19 mg; niacin, 50 mg; folic acid, 1.5 mg; and biotin, 60 μg.

3 Minerals (premix content per kg diet): Co (CoCO_3_), 0.255 mg; Cu (CuSO_4_·5H_2_O), 10.8 mg; Fe (FeSO_4_·H_2_O), 90 mg; Mn (MnSO_4_·H_2_O), 90 mg; Zn (ZnO), 68.4 mg; Se (Na_2_SeO_3_), 0.18 mg.

**Table 2:** Ingredients and calculated analysis (% as fed) of the diet for finisher broilers (22–35 days).

Ingredients^1^	Control	5 % USBH	10 % USBH	5 % FSBH_B_	10 % FSBH_B_
**Composition**	———————————–%———————————				
Corn, yellow	50.01	39.91	28.41	39.71	28.11
Soybean meal (CP-44 %)	21.8	21	9	19.5	8
Full fat soybean meal	20	23	40	25	41.5
Soybean oil	1.6	4.5	6	4.2	5.8
Soybean hull	0	5	10	0	0
Fermented soybean hull	0	0	0	5	10
Fish meal (CP-65 %)	3	3	3	3	3
Monocalcium phosphate	1.3	1.3	1.3	1.3	1.3
Calcium carbonate	1.4	1.4	1.4	1.4	1.4
NaCl	0.34	0.34	0.34	0.34	0.34
_DL_-Methionine	0.27	0.27	0.27	0.27	0.27
Choline-Cl	0.08	0.08	0.08	0.08	0.08
Vitamin premix^2^	0.1	0.1	0.1	0.1	0.1
Mineral premix^3^	0.1	0.1	0.1	0.1	0.1
Total	100	100	100	100	100
**Calculated nutrient levels**					
Crude protein, %	20.03	20.01	20.01	20.06	20.09
ME, kcal/kg	3208	3211	3213	3205	3210
Crude fiber, %	1.98	3.73	4.85	3.64	4.79
Calcium, %	0.92	0.93	0.93	0.93	0.94
Total phosphorus, %	0.62	0.60	0.60	0.61	0.60
Lysine, %	1.11	1.13	1.18	1.14	1.19

1 USBH, unfermented soybean hull; FSBH_B_, fermented soybean hull by *Bacillus velezensis*.

2 Vitamins (premix content per kg diet): Vit. A, 15,000 IU; Vit. D3, 3000 IU; Vit. E, 30 mg; Vit. K3, 4 mg; thiamine, 3 mg; riboflavin, 8 mg; pyridoxine, 5 mg; Vit. B12, 25 μg; Ca-pantothenate, 19 mg; niacin, 50 mg; folic acid, 1.5 mg; and biotin, 60 μg.

3 Minerals (premix content per kg diet): Co (CoCO_3_), 0.255 mg; Cu (CuSO_4_·5H_2_O), 10.8 mg; Fe (FeSO_4_·H_2_O), 90 mg; Mn (MnSO_4_·H_2_O), 90 mg; Zn (ZnO), 68.4 mg; Se (Na_2_SeO_3_), 0.18 mg.

### Growth performance and sample collection

Each group's body weight (**BW**) and feed intake (**FI**), including replicates, were measured on Days 21 and 35. Body weight gain (**BWG**), feed conversion rate (**FCR**), livability rate (**Livability**), and performance efficiency factors (**PEF**) were calculated subsequently. On day 35, six birds with average weight from each group were selected for euthanizing and sampling.

### Quantitative real-time polymerase chain reaction (qPCR) analysis

Jejunum samples soaked in an RNA shield were removed from −20°C and thawed before immersion in 1 mL of RNAzol (Molecular Research Center, Cincinnati, OH, USA). The samples were homogenized, and RNA extraction was performed following the protocol provided by a commercial rapid test kit. The concentration and quality of the extracted RNA were measured. Reverse transcription of the extracted RNA was conducted using a commercial cDNA kit (Applied Biosystems, Waltham, MA, USA), and the cDNA samples were quantified to 300 ng/μL. A mixture of 2X SYBR GREEN PCR Master Mix-ROX (Applied Biosystems, MA, USA), ultrapure water, primers, and the cDNA samples were prepared. This mixture was then analyzed using the StepOnePlus™ Real-Time PCR System (Thermo Fisher, Waltham, MA, USA), and changes in Ct values were recorded. The primer sequences for the target genes were obtained from GenBank and matched the genotype of the *Gallus*. The primer sequences are provided in Table 3.

**Table 3:** Primer sequences of various *Gallus gallus* genes used for quantitative real-time PCR analysis.

Gene name		Primer sequence^1^	Tm^2^	Size^3^	Genbank No.
ß-actin	F: 5′-CTGGCACCTAGCACAATGAA-3′ R: 5′-ACATCTGCTGGAAGGTGGAC-3′		56.4	464	X00182.1
Nrf2	F: 5′-GGAAGAAGGTGCTTTTTCGCAGC-3′ R: 5′-GGGCAAGGCAGATCTCTTCCAA-3′		59.5	116	NM_205117.2
SOD-1	F: 5′-ATTACTGGCTTGTCTGATGG-3′ R: 5′-CCTCCCTTTGCAGTCACATT-3′		54.3	173	NM_205064.2
CAT	F: 5′-CCACGTGGACCTCTTCTTGT-3′ R: 5′-AAACACTTTCGCCTTGCAGT-3′		55.4	165	NM_001031215.2
GSH-Px	F: 5′-AACCAATTCGGGCACCAG- 3′ R: 5′-CCGTTCACCTCGCACTTCTC-3′		57.2	122	NM_001277853.2
HO-1	F: 5′-AGCTTCGCACAAGGAGTGTT-3′ R: 5′-GGAGAGGTGGTCAGCATGTC-3′		57.4	106	X56201.1
NF-κB	F: 5′-GAAGGAATCGTACCGGGAACA-3′ R: 5′-CTCAGAGGGCCTTGTGACAGTAA-3′		58.5	131	D13719.1
iNOS	F: 5′-AGCATAACTCCCGTGTTCCA-3′ R: 5′-GATTTCCCAGTCTCGGTTGC-3′		56.4	239	NM_204961.2
TNF-α	F: 5′-TGTGTATGTGCAGCAACCCGTAGT-3′ R: 5′-GGCATTGCAATTTGGACAGAAGT-3′		57.7	229	NM_204267.2
Muc 2	F: 5′-GCTACAGGATCTGCCTTTGC-3′ R: 5′-AATGGGCCCTCTGAGTTTTT-3′		55.4	152	JX284122.1
ZO-1	F: 5′-AGGTGAAGTGTTTCGGGTTG-3′ R: 5′-CCTCCTGCTGTCTTTGGAAG-3′		56.4	171	XM_015278975.4
OCLN	F: 5′-GTCTGTGGGTTCCTCATCGT-3′ R: 5′-GTTCTTCACCCACTCCTCCA-3′		57.4	156	NM_205128.1
PepT-1	F: 5′-CAGGGATCGAGATGGACACT-3′ R: 5′-CACTTGCAAAAGAGCAGCAG-3′		56.4	243	NM_204365.2

Nrf2, Nuclear factor erythroid 2-related factor 2; SOD-1, Superoxide dismutase 1; CAT, Catalase; GSH-Px, Glutathione peroxidase; HO-1, Heme oxygenase 1; NF-κB, Nuclear factor kappa-light-chain-enhancer of activated B cells p50; iNOS, inducible nitric oxide synthase; TNF-α, Tumor necrosis factor alpha; Muc 2, Mucin 2; ZO-1, Zonula occludens-1; OCLN, Occludin; PepT-1, Peptide transporter 1.

^1,2,3^For each gene the primer sequence for forward and reverse (5′ to 3′), the melting temperature (Tm) in°C, and the product size (Size, bp) are shown.

### Quantitative analysis of cellular antioxidant activity and inflammatory cytokines

Serum and jejunum samples were thawed, and 0.1 g of jejunum sample was immersed in extraction solution and homogenized using a micro stirring mixer on ice. The supernatant obtained after centrifugation was subjected to protein quantification using the Bradford protein assay. Subsequently, commercial ELISA analysis kits were employed for quantitative analysis. Antioxidant activity analysis included superoxide dismutase (**SOD**, Cayman), catalase (**CAT**, Cayman), glutathione peroxidase (**GSH-Px**, Cayman), and malondialdehyde (**MDA**, Cayman). Cellular inflammatory cytokines quantified included tumor necrosis factor alpha (**TNF-α**, Cusabio), interleukin-6 (**IL-6**, FineTest), interleukin-1 beta (**IL-1β**, FineTest), and interleukin-10 (**IL-10**, FineTest).

### Analysis of intestinal microbiota composition

The 16S rRNA gene was utilized to identify species within the intestinal microbiota. Based on previous experimental results, groups exhibiting favorable growth performance were selected for analysis. Specifically, the microbial compositions of the ileum and cecum were compared among the control group, 10 % USBH group, and 10 % FSBH_B_ group. For each group, 3 g of ileum and cecum contents were collected and analyzed by Biotools CO., Ltd using the PacBio Sequel IIe system for full-length 16S amplicon sequencing. Denoising analysis was then performed using a divisive amplicon denoising algorithm, yielding amplicon sequence variants. Species classification analysis included species composition distribution, alpha diversity analysis, beta diversity analysis, and Venn diagrams to assess species richness and evenness within the samples.

### Statistical analysis

The experimental data were analyzed using SAS 9.4M7 software. A one-way analysis of variance (ANOVA) was conducted, and Duncan's new multiple-range test was applied to assess the significance of differences between treatment group means. A threshold of *P* < 0.05 was set to determine statistical significance.

## Results

### Bioactive components

Table 4 illustrates the differences in the phenolic acid components and soybean isoflavones between USBH and FSBH_B_. The FSBHB exhibited an increasing trend in the contents of phenolic acids and isoflavones, with chlorogenic acid and glycitin demonstrating relatively greater increases.

**Table 4:** Comparison of phenolic acid compositions and soy isoflavones in fermented soybean hull.

Item	Treatment^1^	
	USBH	FSBH_B_
	—–phenolic acid compositions (µg/g DM)—–	
Ferulic acid	0.75±0.38	1.28±1.13
p-Coumaric acid	0.96±0.75	1.32±0.20
Chlorogenic acid	7.20±0.88	11.01±0.33
Caffeic acid	0.52±0.10	0.53±0.08
	————soy isoflavones (µg/g DM) ————	
Daidzin	37.9 ± 9.4	55.1 ± 23.1
Glycitin	17.6 ± 4.3	34.8 ± 9.4
Genistin	23.7 ± 6.5	41.1 ± 21.7
Daidzein	9.1 ± 2.8	17.0 ± 6.2
Glycitein	1.1 ± 0.6	5.7 ± 2.1
Genistein	6.2 ± 1.9	8.8 ± 2.7

1 Each value represents the mean with standard deviation (*n* = 3). USBH, unfermented soybean hull; FSBH_B_, fermented soybean hull by *Bacillus velezensis*.

### Growth performance

Table 5 presents the results of growth performance. The groups receiving 5 % USBH, 10 % USBH, 5 % FSBH_B_, and 10 % FSBH_B_ had significantly higher (*P* < 0.05) BW, FI, and BWG during the early rearing period compared to the control group, with no differences observed between the soybean hull groups. During the later rearing period, the soybean hull groups exhibited increased FI, resulting in a significantly lower (*P* < 0.05) FCR compared to the control group. Across the entire rearing period, FI was significantly higher (*P* < 0.05) in the soybean hull groups than in the control group. However, the control group demonstrated better FCR performance, though no significant differences (*P* > 0.05) were observed compared to the 10 % FSBH_B_ group.

**Table 5:** Effect of dietary substitution with different levels of fermented soybean hull on growth performance of broilers.

Experimental period (d)	Experimental diet^1^					SEM^2^	*P*-value
	Control	5 % USBH	10 % USBH	5 % FSBH_B_	10 % FSBH_B_		
	————— Body weight, BW (g) —————						
1-21	852.2^b^	954.2^a^	938.7^a^	939.2^a^	928.6^a^	16.5	<0.05
22-35	2118.0	2200.2	2213.5	2209.6	2264.2	57.0	0.51
	————— Feed intake, FI (g) —————						
1-21	1034.4^b^	1138.1^a^	1113.1^a^	1130.6^a^	1110.3^a^	19.5	<0.05
22-35	1777.4	1965.8	1951.7	2007.0	2005.4	59.2	0.08
1-35	2811.8^b^	3103.9^a^	3064.8^a^	3137.6^a^	3115.7^a^	70.2	<0.05
	———- Body weight gain, BWG (g) ———-						
1-21	813.6^b^	915.7^a^	900.4^a^	900.8^a^	890.5^a^	16.5	<0.05
22-35	1265.8	1246.0	1259.6	1268.8	1310.0	47.3	0.90
1-35	2079.4	2161.7	2160.1	2169.6	2200.5	53.9	0.61
	———- Feed conversion ratio, FCR ———-						
1-21	1.27	1.24	1.24	1.26	1.25	0.01	0.47
22-35	1.41^b^	1.58^a^	1.55^a^	1.59^a^	1.53^a^	0.04	<0.05
1-35	1.35^b^	1.44^a^	1.42^ab^	1.45^a^	1.42^ab^	0.02	<0.05
	—————— Livability (%) ——————						
	97.5	100.0	96.3	97.5	96.3	1.8	0.57
	—– Performance efficiency factor, PEF^3^ —–						
	436.3	438.0	429.3	426.7	440.0	17.5	0.98

^1^Each value represents the mean of 4 replicates (20 birds in each replicate). USBH, unfermented soybean hull; FSBH_B_, fermented soybean hull by *Bacillus velezensis*.

^2^SEM: Standard error of the mean.

^3^Performance efficiency factor = [(BW(kg) × Livability (%)) ÷ (FCR × Days of age)] × 100.

^a,b^Means within the same row with different letters are significantly different (*P* < 0.05).

### Expression of antioxidant regulatory genes

**Fig. 1:**
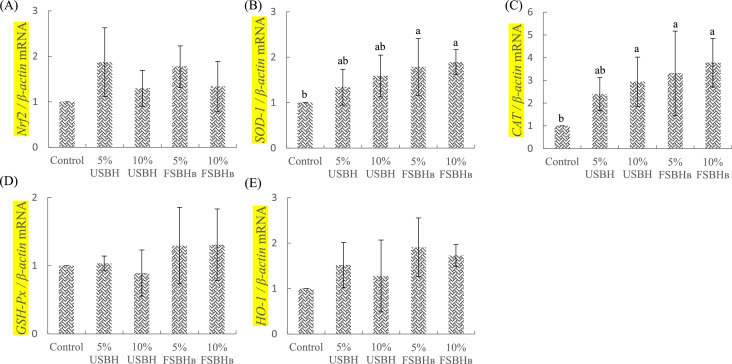
Effect of dietary substitution with different levels of fermented soybean hull on jejunum's relative mRNA expression of genes related to Nrf2 (A), SOD-1 (B), CAT (C), GSH-Px (D), and HO-1 (E) of 35d-old broilers. ^a, b^ Different letters are significantly different (*P* < 0.05), each value represent mean of 4 replicates. Error bars represent the SD of the mean.

Fig. 1 (A-E) displays the expression levels of antioxidant genes in the jejunum of broilers. In Fig. 1(B), the expression of the SOD-1 gene was significantly elevated (*P* < 0.05) in the 5 % FSBH_B_ and 10 % FSBH_B_ groups compared to the control group. Similarly, Fig. 1(C) indicates that CAT gene expression was notably higher (*P* < 0.05) in the 10 % USBH, 5 % FSBH_B_, and 10 % FSBH_B_ groups than in the control group. However, no statistically significant differences (*P* > 0.05) were observed in the expression levels of nuclear factor erythroid 2-related factor 2 (**Nrf2**), GSH-Px, and heme oxygenase 1 (**HO-1**) genes across the groups.

### Expression of inflammatory cytokine genes

**Fig. 2:**
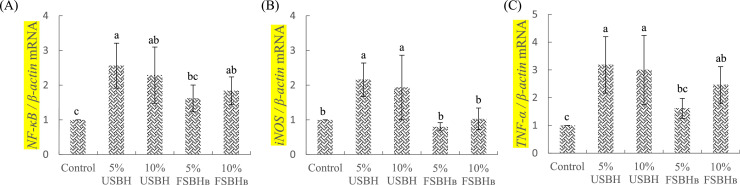
Effect of dietary substitution with different levels of fermented soybean hull on jejunum's relative mRNA expression of genes related to NF-κB (A), iNOS (B), and TNF-α (C) of 35d-old broilers. ^a-c^ Different letters are significantly different (*P* < 0.05), each value represent mean of 4 replicates. Error bars represent the SD of the mean.

Fig. 2 (A-C) depicts the expression levels of inflammatory cytokine genes in the jejunum of broilers. In Fig. 2(A), no significant differences (*P* > 0.05) were observed in nuclear factor kappa-light-chain-enhancer of activated B cells (**NF-κB**) gene expression between the control group and the 5 % FSBH_B_ group. Significant differences (*P* < 0.05) were found between the control group and the 5 % USBH group, 10 % USBH group, and 10 % FSBH_B_ group. Fig. 2(B) reveals significant differences (*P* < 0.05) in the expression of the inducible nitric oxide synthase (**iNOS**) gene among the control group, 5 % USBH group, and 10 % USBH group. In Fig. 2(C), no significant differences (*P* > 0.05) were detected in TNF-α gene expression between the control group and the 5 % FSBH_B_ group. Additionally, there were significant differences (*P* < 0.05) in expression levels between the control group and the 5 % USBH group, 10 % USBH group, and 10 % FSBH_B_ group.

### Expression of mucin and tight junction genes

**Fig. 3:**
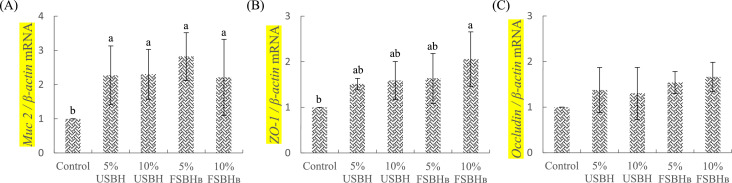
Effect of dietary substitution with different levels of fermented soybean hull on jejunum's relative mRNA expression of genes related to Muc 2 (A), ZO-1 (B), and Occludin (C) of 35d-old broilers. ^a, b^ Different letters are significantly different (*P* < 0.05), each value represent mean of 4 replicates. Error bars represent the SD of the mean.

The expression of tight junction genes in the jejunum of broilers is illustrated in Fig. 3 (A-C). Fig. 3(A) shows that mucin 2 (**Muc 2**) gene expression levels were significantly higher (*P* < 0.05) in all soybean hull treatment groups compared to the control group. Fig. 3(B) demonstrates that zonula occludens-1 (**ZO-1**) gene expression was significantly higher (*P* < 0.05) in the 10 % FSBH_B_ group than in the control group. In Fig. 3(C), the expression levels of the occludin gene did not show any statistically significant differences (*P* > 0.05) between the groups.

### Expression of PepT1 genes in jejunum epithelial cells

**Fig. 4:**
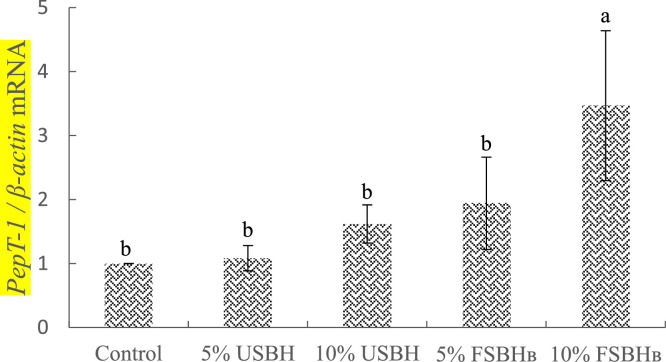
Effect of dietary substitution with different levels of fermented soybean hull on jejunum's relative mRNA expression of genes related to PepT-1 of 35d-old broilers. ^a, b^ Different letters are significantly different (*P* < 0.05), each value represent mean of 4 replicates. Error bars represent the SD of the mean.

The expression of genes related to peptide transporter 1 in the jejunum of broilers is illustrated in Fig. 4. The expression levels of the peptide transporter 1 (**PepT-1**) gene were significantly higher (*P* < 0.05) in the 10 % FSBH_B_ group compared to both the control group and the other treatment groups.

### Expression of serum and intestinal antioxidant proteins

Table 6 presents the expression of antioxidant proteins in serum and jejunum. In the jejunum, no statistically significant differences (*P* > 0.05) were observed in the levels of SOD, CAT, GSH-Px, and MDA. In serum, SOD activity was significantly higher (*P* < 0.05) in the 5 % FSBH_B_ and 10 % FSBH_B_ groups compared to the control, 5 % USBH, and 10 % USBH groups, with the highest activity observed in the 10 % FSBH_B_ group. For GSH-Px, the protein expression level was significantly higher (*P* < 0.05) in the 5 % FSBH_B_ group compared to the control group. No significant differences (*P* > 0.05) were observed among the 10 % USBH, 5 % FSBH_B_, and 10 % FSBH_B_ groups. Similarly, no statistically significant differences (*P* > 0.05) were found in CAT and MDA levels in serum.

**Table 6:** Effect of dietary substitution with different levels of fermented soybean hull on serum, jejunum’ s antioxidant activity of 35d-old broilers.

Item	Experimental diet^1^					SEM^2^	*P*-value
	Control	5 % USBH	10 % USBH	5 % FSBH_B_	10 % FSBH_B_		
Jejunum							
SOD (U/mg protein)	242.2	242.5	249.3	253.0	242.6	14.1	0.97
CAT (nmol/min/mg protein)	39.0	35.3	45.9	52.0	52.5	8.6	0.54
GSH-Px (nmol/min/mg protein)	39.7	43.0	36.2	43.4	36.2	2.7	0.18
MDA (μM)	26.5	31.2	26.2	25.0	23.8	4.3	0.79
Serum							
SOD (U/mg protein)	5.21^c^	5.71^c^	6.81^c^	9.09^b^	12.20^a^	0.54	<0.05
CAT (nmol/min/mg protein)	0.68	0.65	0.67	0.56	0.81	0.09	0.45
GSH-Px (nmol/min/mg protein)	8.76^b^	8.36^b^	9.78^ab^	10.45^a^	9.35^ab^	0.50	0.05
MDA (μM)	42.0	31.3	30.9	30.4	33.9	4.3	0.31

SOD, Superoxide dismutase; CAT, Catalase; GSH-Px, Glutathione peroxidase; MDA, Malondialdehyde.

^1^Each value represents the mean of 6 replicates. USBH, unfermented soybean hull; FSBH_B_, fermented soybean hull by *Bacillus velezensis*.

^2^SEM: Standard error of the mean.

^a-c^Means within the same row with different letters are significantly different (*P* < 0.05).

### Expression of intestinal inflammatory cytokine proteins

Table 7 presents the protein expression of inflammatory cytokines in the jejunum. For TNF-α, the protein expression level in the 10 % FSBH_B_ group was significantly lower (*P* < 0.05) than that in the control group, whereas the protein expression level in the 5 % USBH group was significantly higher (*P* < 0.05) than that in the control group. However, no significant differences (*P* > 0.05) were observed in the protein expression levels among the control group, the 10 % USBH group, and the 5 % FSBH_B_ group. Additionally, no statistically significant differences (*P* > 0.05) were found in the protein expression levels of IL-6, IL-1β, and IL-10.

**Table 7:** Effect of dietary substitution with different levels of fermented soybean hull on jejunum's immunity of 35d-old broilers.

Item	Experimental diet^1^					SEM^2^	*P*-value
	Control	5 % USBH	10 % USBH	5 % FSBH_B_	10 % FSBH_B_		
————————- pg/mg protein ————————-							
TNF-α	1.6^b^	2.6^a^	2.1^ab^	1.6^b^	0.6^c^	0.30	<0.05
IL-6	1376.6	1557.4	1697.8	1618.6	1786.6	300.1	0.89
IL-1β	673.5	631.4	718.4	732.5	831.6	134.9	0.86
IL-10	270.4	209.0	264.3	346.7	332.9	63.0	0.56

^1^Each value represents the mean of 3 replicates. USBH, unfermented soybean hull; FSBH_B_, fermented soybean hull by *Bacillus velezensis*.

^2^SEM: Standard error of the mean.

^a-c^Means within the same row with different letters are significantly different (*P* < 0.05).

### Intestinal microbiota composition

**Fig. 5:**
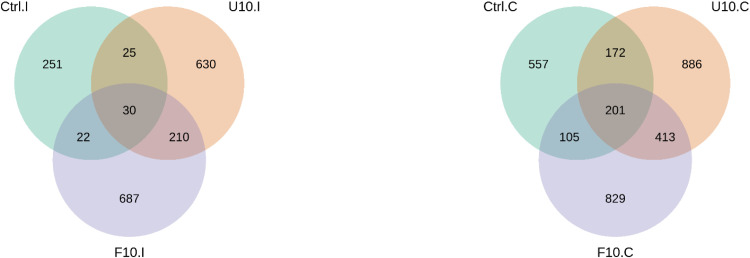
Venn diagram for microbiota of broilers in ileum (A) and cecum (B). Ctrl: basal diet; U10: partially replaced control with 10 % of unfermented soybean hull; F10: partially replaced control with 10 % of fermented soybean hull by *Bacillus velezensis*.

Figs. 5 to 9 depicted the impact of substituting the basal diet with unfermented and fermented soybean hulls on the microbiota composition in the ileum and cecum of broilers. Fig. 5 (A, B) shows the microbial Venn diagrams for broilers. In Panel (A), it was observed that in the ileum, the 10 % USBH group and the 10 % FSBH_B_ group each had over 600 different microbial species compared to the control group. They shared a total of 210 microbial species that differed from those in the control group. In Panel (B), it was noted that both the 10 % USBH group and the 10 % FSBH_B_ group exhibited over 800 distinct microbial species in the cecum compared to the control group. Additionally, they collectively shared 413 microbial species that differed from those observed in the control group.

**Fig. 6.1:**
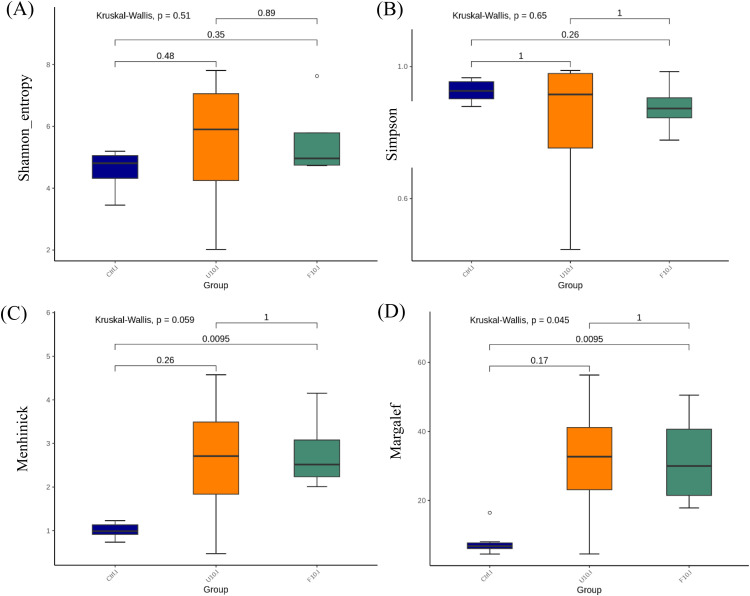
Alpha diversity indices of the bacterial community in the ileum of broilers. (A) Shannon index, (B) Simpson index, (C) Menhinick's richness index, and (D) Margalef's richness index. Significance was assessed by Kruskal-Wallis. Ctrl: basal diet; U10: partially replaced control with 10 % of unfermented soybean hull; F10: partially replaced control with 10 % of fermented soybean hull by *Bacillus velezensis*.

**Fig. 6.2:**
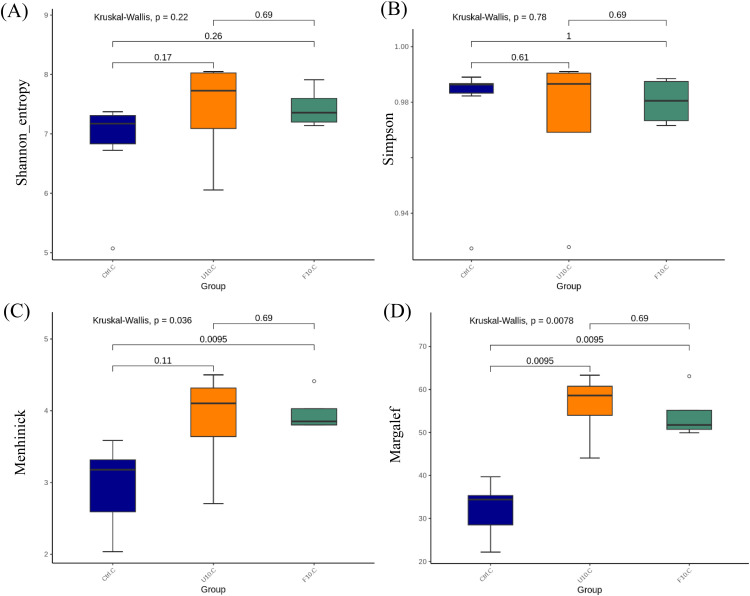
Alpha diversity indices of the bacterial community in the cecum of broilers. (A) Shannon index, (B) Simpson index, (C) Menhinick's richness index, and (D) Margalef's richness index. Significance was assessed by Kruskal-Wallis. Ctrl: basal diet; U10: partially replaced control with 10 % of unfermented soybean hull; F10: partially replaced control with 10 % of fermented soybean hull by *Bacillus velezensis*.

Fig. 6.1, Fig. 6.2 (A-D) present the alpha-diversity indices of intestinal microbiota in broilers, including species evenness (Shannon and Simpson indices) and species richness (Menhinick and Margalef indices), which were used to assess species diversity. In Fig. 6.1 (A, B), no statistically significant differences (*P* > 0.05) were observed in the evenness indices among the groups in the broiler ileum. In Fig. 6.1 (C, D), the Margalef index was significantly higher (*P* < 0.05) in the 10 % FSBH_B_ group compared to the control group. However, the Menhinick index showed no statistical differences (*P* > 0.05), though there was a trend towards higher values in the 10 % FSBH_B_ group. Differences in richness indices may arise from calculation methods; Menhinick calculates based on the number of species in the sample, and Margalef subtracts one from the species count, amplifying differences in smaller samples. In Fig. 6.2 (A, B), no statistically significant differences (*P* > 0.05) were observed in the evenness indices among the groups in broilers cecum. However, in Fig. 6.2 (C, D), the Menhinick index was significantly higher (*P* < 0.05) in the 10 % FSBH_B_ group compared to the control group. Both the 10 % USBH and 10 % FSBH_B_ groups showed significantly higher (*P* < 0.05) Margalef indices compared to the control group.

**Fig. 7.1:**
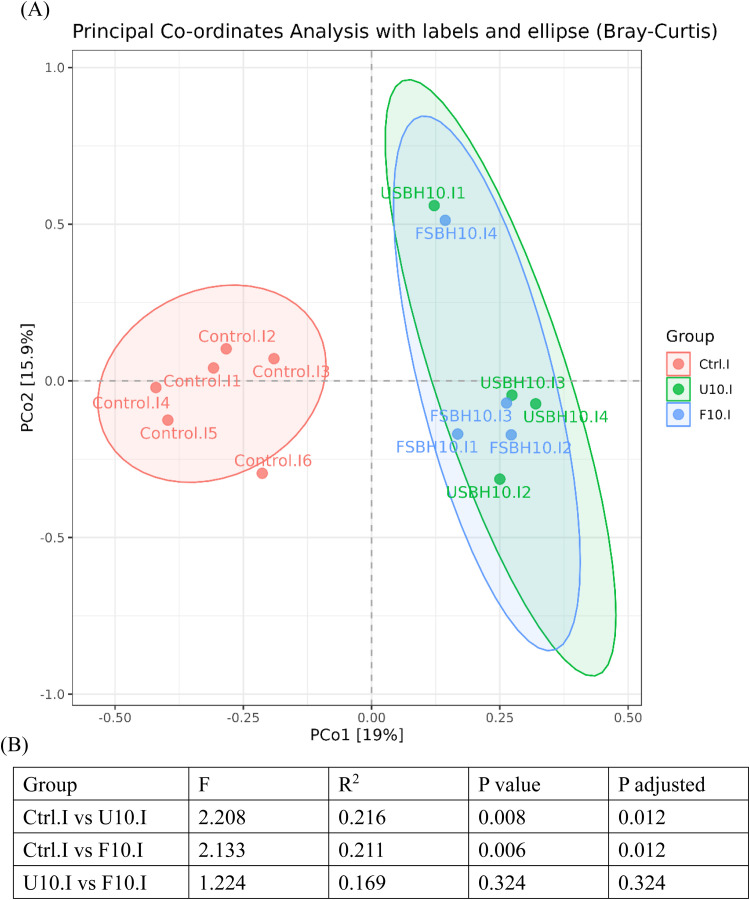
Beta diversity indices of the bacterial community in the ileum of broilers. (A) PCoA analysis, and (B) PERMANOVA. Ctrl: basal diet; U10: partially replaced control with 10 % of unfermented soybean hull; F10: partially replaced control with 10 % of fermented soybean hull by *Bacillus velezensis*. F: measure R²: the degree to which different groupings explain the variation in the samples, defined as the ratio of group variance to total variance. The adjusted p-value being less than 0.05 indicates high reliability of this test.

**Fig. 7.2:**
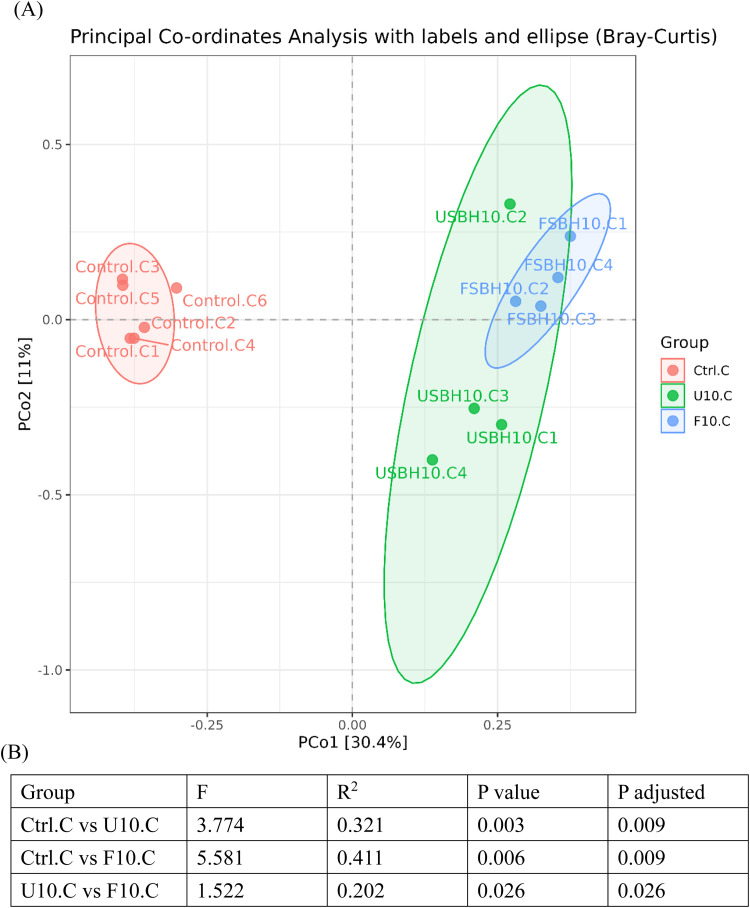
Beta diversity indices of the bacterial community in the cecum of broilers. (A) PCoA analysis, and (B) PERMANOVA. Ctrl: basal diet; U10: partially replaced control with 10 % of unfermented soybean hull; F10: partially replaced control with 10 % of fermented soybean hull by *Bacillus velezensis*. F: measure R²: the degree to which different groupings explain the variation in the samples, defined as the ratio of group variance to total variance. The adjusted p-value being less than 0.05 indicates high reliability of this test.

In Fig. 7.1(A), the ileum's PCoA analysis (beta diversity) showed that PCo1 and PCo2 explained 19 % and 15.9 % of the total variance, respectively. The control group samples were primarily distributed on the left side of the plot, while the USBH and FSBH group samples were positioned on the right side. In Fig. 7.1(B), the PERMANOVA test further confirmed a significant difference in the microbial community structure between the control group and the USBH group (R² = 0.216, *p* < 0.05). A significant difference was also observed between the control group and the FSBH group (R² = 0.211, *p* < 0.05). In Fig. 7.2(A), the PCoA analysis (beta diversity) of the cecum revealed that PCo1 and PCo2 explained 30.4 % and 11 % of the total variance, respectively. Similar to the results from the ileum, the control group samples were primarily distributed on the left side of the plot, while the USBH and FSBH group samples were mainly positioned on the right side. In Fig. 7.2(B), the PERMANOVA test further confirmed significant differences in the microbial community structure between the control group and the USBH group (R² = 0.321, *p* < 0.05), as well as between the control group and the FSBH group (R² = 0.411, *p* < 0.05). A significant difference was also observed between the USBH and FSBH groups (R² = 0.202, *p* < 0.05).

**Fig. 8:**
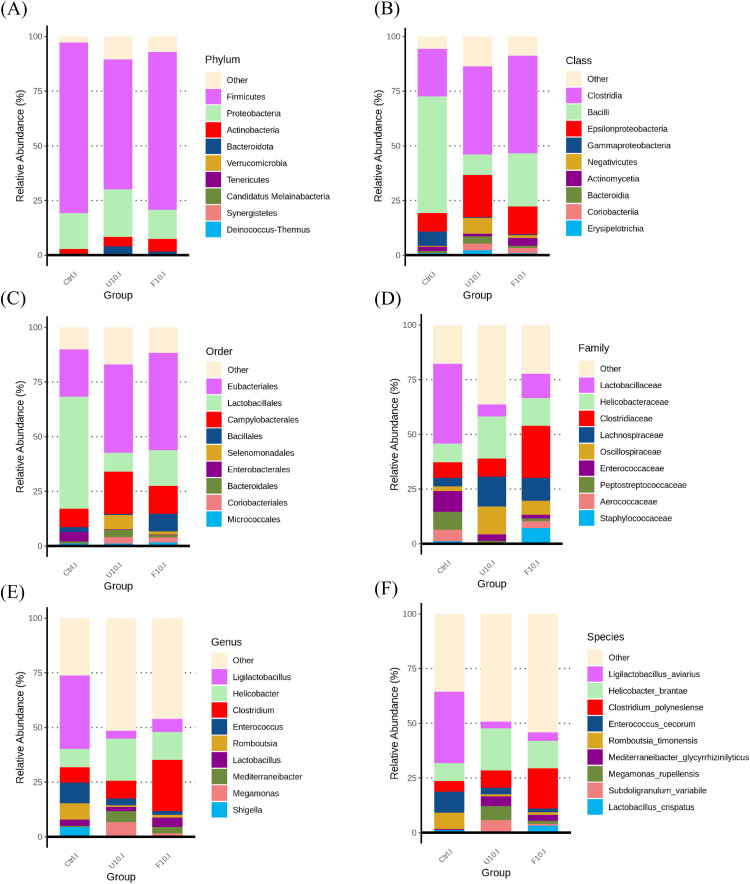
100 % stacked bar chart of the top 10 with the highest relative abundance in ileum under (A) phylum, (B) class, (C) order, (D) family, (E) genus and (F) species. Ctrl: basal diet; U10: partially replaced control with 10 % of unfermented soybean hull; F10: partially replaced control with 10 % of fermented soybean hull by *Bacillus velezensis*.

Fig. 8 (A-F) shows the relative abundance of the top 10 species from phylum to species level in the microbiota of broiler ileum. At the phylum level (A), there were no significant differences in relative abundance among the groups. At the class level (B), both the 10 % USBH and 10 % FSBH_B_ groups exhibited a decreasing trend in the class *Bacilli*, with the 10 % USBH group showing the largest decrease. This decrease was accompanied by increases in the classes *Clostridia* and *Epsilonproteobacteria*. At the order level (C), there was a decrease in the order *Lactobacillales* in both groups, offset by increases in the orders *Eubacteriales* and *Campylobacterales*, with the 10 % FSBH_B_ group also showing an increase in the order *Bacillales*. At the family level (D), both groups exhibited a decrease in the *Lactobacillaceae*, while *Helicobacteraceae, Clostridiaceae*, and *Lachnospiraceae* showed varying degrees of increase. At the genus level (E), there was a notable decrease in the genus *Ligilactobacillus* in both groups. In contrast, the genera *Helicobacter* and *Clostridium* showed an increase. Additionally, the genus *Lactobacillus* demonstrated a slight increase in the 10 % FSBH_B_ group. At the species level (F), there was a decrease in *Ligilactobacillus aviarius* and *Enterococcus cecorum* in both groups. However, *Helicobacter brantae* and *Clostridium polynesiense* showed an increase.

**Fig. 9:**
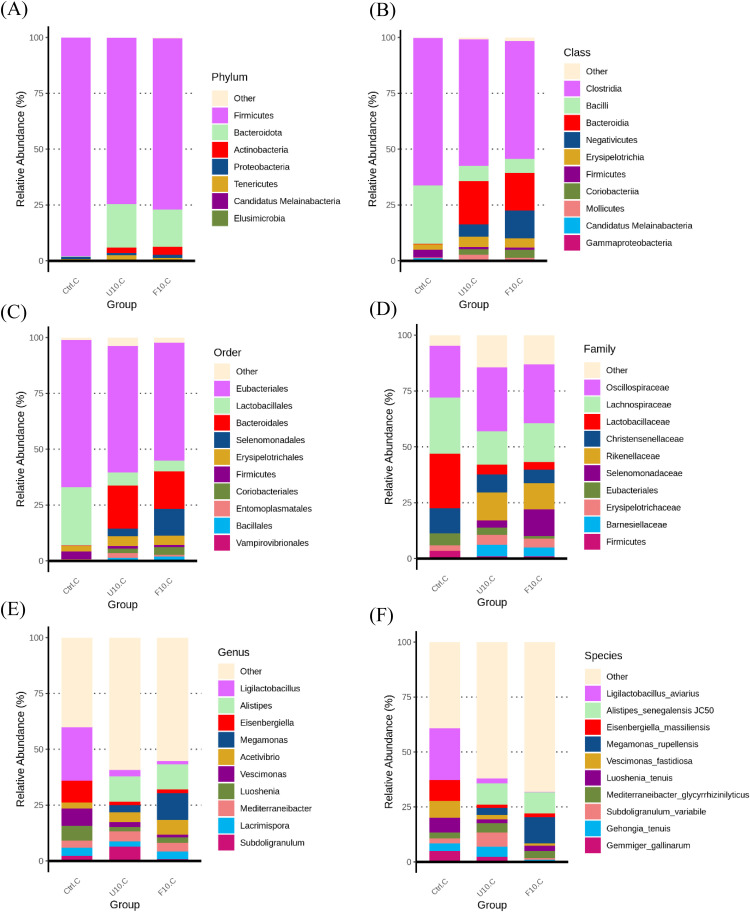
100 % stacked bar chart of the top 10 with the highest relative abundance in cecum under (A) phylum, (B) class, (C) order, (D) family, (E) genus and (F) species. Ctrl: basal diet; U10: partially replaced control with 10 % of unfermented soybean hull; F10: partially replaced control with 10 % of fermented soybean hull by *Bacillus velezensis*.

Fig. 9 (A-F) depicts the relative abundance of the top 10 species from the phylum to species level in the microbiota of broiler cecum. At the phylum level (A), both the 10 % USBH and 10 % FSBH_B_ groups showed a decreasing trend in the phyla *Firmicutes/Bacteroidota* ratio. At the class level (B), both groups exhibited a decrease in the *Bacilli*, which was compensated by increases in the *Bacteroidia* and *Negativicutes*. At the order level (C), there was a decrease in the order *Lactobacillales* in both groups. However, the *Bacteroidales* and *Selenomonadales* displayed an increasing trend. At the family level (D), there was a decrease in the family *Lactobacillaceae* in both groups, whereas the families *Rikenellaceae* and *Selenomonadaceae* demonstrated an increase. At the genus level (E), there was a decrease in the genera *Ligilactobacillus* and *Eisenbergiella* in both groups, with an increase observed in the genera *Alistipes* and *Megamonas*. At the species level (F), both groups revealed a decrease in *Ligilactobacillus aviarius* and *Eisenbergiella massiliensis*, with *Alistipes senegalensis* JC50 and *Megamonas rupellensis* showing an increase.

## Discussion

This study replaced the unfermented soybean hulls (5 % USBH, 10 % USBH) and fermented soybean hulls (5 % FSBHB, 10 % FSBHB) in the diet with 5 % and 10 % concentrations, respectively. The results indicated that the appropriate use of soybean hulls fermented by *Bacillus velezensis* can increase soluble peptides and improve broiler chickens’ growth, intestinal morphology and antioxidant activity. González-Alvarado et al. (2007) found that adding 3 % oat hulls or 3 % soybean hulls to broiler diets improved BWG and FI while reducing FCR in 14 to 21-day-old broilers. They also found that adding an appropriate amount of hulls could lower gizzard pH and increase nutrient utilization, thereby improving early production performance in broilers. Another study by Tejeda and Kim (2021) showed that a 4 % soybean hull group (replacing 5.62 %) performed better in FCR during the first 7 days of broiler age and improved the villus height to crypt depth (VH/CD) ratio of the jejunum. However, the 8 % soybean hull group (replacing 17.77 %) increased intestinal viscosity, which reduced growth performance and increased mortality.

The Keap1-Nrf2 pathway is a crucial regulatory mechanism in the antioxidant system. Under oxidative stress, the activity of Keap1 is reduced, allowing Nrf2 to enter the nucleus and bind to the ARE or EpRE sequences on DNA, initiating the transcription of downstream genes. This results in the synthesis of antioxidant-related enzymes such as SOD, GSH-Px, CAT, GR, GCL, Trx, TrxR, and HO-1, which help prevent damage caused by oxidative stress and inflammation (Kouvedaki et al., 2024; Canning et al., 2015). According to the research by Tonolo et al. (2020), bioactive peptides produced from fermented soybeans after simulated gastrointestinal digestion exhibited antioxidant capacity in vitro and in cell models. They activated the Keap1/Nrf2 pathway, inducing the expression of antioxidant genes, presumably because the peptides bound to the Kelch domain of Keap1, reducing its inhibitory effect on Nrf2. Yi et al. (2020) found that low molecular weight soybean peptides could protect HepG2 cells from H_2_O_2_-induced oxidative damage and regulate the gene and protein expression of CAT, GSH-Px, and SOD. This was due to the peptides decreasing Keap1 expression, further reducing Nrf2 ubiquitination, allowing Nrf2 to regulate antioxidant enzyme activity and ultimately inhibiting the production of ROS and MDA. Theoretically, fermentation of soybean protein produces various types of peptides, which may regulate different target proteins through different pathways based on the functional groups present in amino acids. However, the experimental results showed no difference in Nrf2 expression between the USBH and FSBH groups, although the FSBH group exhibited an upward trend in the expression of antioxidant-related enzymes, with some significantly higher than the control group. On the other hand, Jiang et al. (2007) found that adding soybean isoflavones to male broilers aged 43-63 days increased the activity of antioxidant enzymes in plasma and muscle, improved growth performance, and prevented MDA production. This was because soybean isoflavones inhibited the formation of lipid oxidation products in the body (Kim, 2021). Given the higher content of phenolic compounds in the FSBH group, and considering that phenolic compounds are recognized as indicators of antioxidant capacity with free radical scavenging ability, as described by Kruk et al. (2022), they could regulate the Nrf2 pathway, stimulating the expression of antioxidant enzymes to reduce ROS formation. Therefore, the bioactive components produced from the fermentation of soybean hulls may have prevented oxidative stress in broilers. Additionally, the USBH group, through its bioactive components, regulated the mRNA expression of Nrf2-related genes while simultaneously reducing the expression of oxidative stress-related genes, thereby enhancing the antioxidant status of broilers.

An inflammatory response may be triggered when tissues are exposed to harmful substances, leading to tissue damage. Common inflammatory pathways include NF-κB, mitogen-activated protein kinase (**MAPK**), and janus kinase-signal transducer and activator of transcription. Toll-like receptors recognize foreign substances and are responsible for transmitting cellular signals, which stimulate transcription factors to enter the nucleus and bind to specific DNA sequences. This ultimately results in the production of pro-inflammatory cytokines such as IL-1β, IL-6, and TNF-α. Concurrently, cells secrete anti-inflammatory cytokines like interleukin-4 and IL-10 to regulate the immune response (Chen et al., 2017). According to Das et al. (2022), fermented soybean foods contain various functional components such as isoflavones, peptides, amino acids, and vitamins, which can participate in inflammatory pathways and regulate cellular signaling molecules. In an in vitro cell model, lipopolysaccharide (**LPS**)-induced inflammation in RAW264.7 cells was reduced by adding soybean peptides. This was achieved by inhibiting the activity of lymphocyte antigen 96, thereby reducing the binding of LPS to toll-like receptor 4 (**TLR4**) and subsequently preventing the expression of the NF-κB and MAPK pathways. As a result, the release of pro-inflammatory cytokines (TNF-α, IL-6, and IL-1β) was inhibited, preventing LPS-induced inflammation in RAW264.7 cells (Yi et al., 2020). Lee et al. (2014) revealed that specific peptides and daidzein in fermented soybean extracts could inhibit LPS-induced iNOS activity and suppress iNOS mRNA expression, thus reducing NO synthesis from arginine and preventing cellular damage. Additionally, genistein could inhibit NF-κB, thereby suppressing the expression of IL-1β and TNF-α (Kouvedaki et al., 2024). Daidzein could inhibit the expression of TLR4 and Myeloid differentiation primary response 88 proteins, as well as NF-κB activity, by reducing the expression of TNF-α and IL-6 (Kim, 2021). According to the results of this study, there were no significant differences in the expression of NF-κB, TNF-α, and iNOS between the control group and the 5 % FSBH_B_ group. Compared to the USBH group, the bioactive ingredients produced from fermentation regulated the NF-κB pathway in broilers.

The intestinal barrier consists of a mucus layer, intestinal epithelial cells, tight junctions (**TJs**), and the lamina propria. The mucus layer prevents intestinal damage and pathogen invasion while providing an appropriate environment for commensal bacteria, promoting nutrient absorption. TJs, composed of ZO-1, Occludin (**OCLN**), Claudin (**CLDN**), and Actin-Myosin cytoskeletal proteins, form barriers between epithelial cells and regulate intestinal permeability (Proszkowiec-Weglarz et al., 2020). Studies by Liu et al. (2024), Song et al. (2022), and He et al. (2016) showed that phenolic compounds (such as ferulic acid, chlorogenic acid, etc.) regulated the expression of TJs proteins (such as Claudin, Occludin, and ZO-1) by inhibiting LPS expression, preventing damage to the intestinal barrier. These studies indicate that phenolic compounds effectively prevent TJ structure damage, thereby maintaining the integrity of the intestinal barrier. Song et al. (2022) noted that phenolic compounds containing a benzene ring with two or more hydroxyl groups contributed to their effectiveness in reducing oxidative stress and improving intestinal inflammation.

Selle et al. (2020) and Poncet and Taylor (2013) explained that nutrient absorption in the intestinal lumen is crucial for the production performance of broilers. When proteins are broken down into single amino acids, dipeptides, and tripeptides, they are absorbed by intestinal epithelial cells via transport proteins. Dipeptides and tripeptides are primarily absorbed through the PepT-1, with small peptide fragments having higher availability for regulating intestinal antioxidant and immune functions compared to single amino acids. Zhu et al. (2020) indicated that PepT-1 alleviates intestinal inflammation by absorbing bioactive peptides derived from soybean protein.

The gut microbiota of poultry plays a crucial role in influencing feed digestion and nutrient absorption, thus closely affecting growth performance and gut health. Under environmental stress, heat stress, and varying rearing conditions, the gut microbiota helps protect the intestines from damage and prevents a decline in growth performance. Due to their strong antioxidant capacity, polyphenols can be used to improve gut health. The interaction between polyphenols and gut microbiota produces bioactive metabolites that enhance the bioavailability of polyphenols and regulate the composition of the gut microbiota (Iqbal et al., 2020). Peptides can produce metabolites with antioxidant activity, such as GSH, folic acid, and butyrate, by regulating the gut microbiota, thereby protecting cells from oxidative damage by scavenging ROS (Wu et al., 2021). The cecum microbiota maintains gut health and stabilizes microbial communities by fermenting chyme, degrading indigestible starch, and producing short-chain fatty acids (**SCFAs**), which help reduce foodborne pathogens like *Salmonella*.

In the poultry gut, the dominant phyla are *Firmicutes, Bacteroidetes, Proteobacteria, Actinobacteria,* and *Tenericutes* (Feye et al., 2020). The ratio of *Firmicutes* to *Bacteroidetes* is related to energy production efficiency, with *Firmicutes* involved in polysaccharide degradation and butyrate production and *Bacteroidetes* responsible for degrading complex carbohydrates and synthesizing propionate (Zhu et al., 2019). The genus *Alistipes*, a major member of the Rikenellaceae family within the Bacteroidales order, produces succinate, which enters the citric acid cycle and helps synthesize glucose, thus benefiting the host gut (Abe et al., 2012). The families *Lachnospiraceae, Ruminococcaceae*, and *Erysipelotrichaceae* degrade plant materials and produce SCFAs, improving FCR and BWG in broilers (Biddle et al., 2013; Stanley et al., 2016). *Lactobacillus* produces lactic acid, which can be converted into SCFAs by *Clostridium lactatifermentans* and serves as an energy source in the gut (Stanley et al., 2016). Li et al. (2022) found that soybean hull insoluble dietary fiber is a good carbon source for gut microbiota growth. In this study, soybean hull insoluble dietary fiber inhibited the growth of *Firmicutes* and promoted the proliferation of *Bacteroidetes* compared to the control group, maintaining microbial balance and promoting the growth of *Lactobacillus* and *Bifidobacterium*. The gut microbiota improves broiler growth performance by metabolizing nutrients such as polysaccharides, lipids, vitamins, and amino acids and regulating gut immune function.

## Conclusions

In terms of intestinal gene regulation, the FSBH group demonstrated better improvement in the gene expression of antioxidant enzymes and suppression of pro-inflammatory cytokines compared to the USBH group. Specifically, the 5 % FSBH_B_ group showed a tendency to suppress pro-inflammatory genes, while the 10 % FSBH_B_ group tended to promote antioxidant genes. Additionally, the high expression of peptide transport genes positively influenced nutrient absorption and the bioactivity of peptides, contributing to broilers’ growth and intestinal health. Therefore, as a functional feed ingredient, fermented soybean hulls could partially replace corn and soybean meal, thereby enhancing the value and feasibility of using soybean hulls in broiler feed.

## Data availability statement

The data presented in this study are available on request from the corresponding author upon reasonable request.

## CRediT authorship contribution statement

**Yung Hao Chen:** Conceptualization, Data curation, Formal analysis, Software. **Yi Chen Li:** Data curation, Resources, Visualization. **Shen Chang Chang:** Formal analysis, Software. **Min Jung Lin:** Investigation. **Li Jen Lin:** Methodology, Validation. **Tzu Tai Lee:** Funding acquisition, Project administration, Supervision, Writing – original draft, Writing – review & editing.

## Declaration of competing interest

The authors declare that they have no known competing financial interests or personal relationships that could have appeared to influence the work reported in this paper.
